# Switched after Birth: Performance of the Viburnum Leaf Beetle [*Pyrrhalta viburni* (Paykull)] after Transfer to a Suboptimal Host Plant

**DOI:** 10.3390/insects5040805

**Published:** 2014-10-27

**Authors:** Gaylord A. Desurmont, Paul A. Weston

**Affiliations:** Department of Entomology, Cornell University, Ithaca, NY 14850, USA

**Keywords:** insect-plant interactions, invasive pest, oligophagous herbivore, diet mixing

## Abstract

Host plant switching is common among phytophagous insects. Once optimal food sources have been depleted, immature insects may resort to use of suboptimal hosts in order to complete their development. Such host switching may have dramatic consequences for insect fitness. Here we investigate the effects of host switching in larvae of the viburnum leaf beetle, *Pyrrhalta viburni*, an invasive landscape pest in North America. Specifically, we examine how transfer of 3rd instar larvae from the optimal host *Viburnum dentatum* to three suboptimal hosts (*V. lentago*, *V. carlesii*, and *V sieboldii*) affects larval development and survivorship to the adult stage. Larval survivorship, pupal weight, and adult weight were overall lower for *P. viburni* larvae that switched hosts, independently of the suboptimal host tested. This decrease in performance corresponds to a decreased feeding rate on suboptimal hosts. Subsequent choice tests showed that 3rd instar larvae become less choosy as they approach pupation, and discriminate less between optimal and suboptimal hosts past a certain weight threshold. In conclusion, *P. viburni* larvae are able to complete their development on suboptimal hosts, but host switching negatively impacts several fitness correlates. Mixed ornamental gardens containing both optimal and suboptimal Viburnum species may provide to outbreaking *P. viburni* populations opportunities to survive the depletion of their preferred food sources.

## 1. Introduction

Dietary breadth is a key component of the ecology, evolution, and diversification of phytophagous insects [1,2]. Classically, insect herbivores are grouped into three categories depending on the range of foods they can consume: monophagous insects only feed and develop in nature on one (or a few closely related) plant species, oligophagous insects feed on a number of species belonging to one (or a few) families, and polyphagous insects feed on a wide range of plant species belonging to many families [3]. However, most phytophagous insects, even among polyphagous species, complete their development on a single plant, the one where eggs were deposited [4]. Host switching occurs when insects move from the plant they were originally feeding on to a conspecific plant (intraspecific host switching) [5] or to a plant belonging to a different species (interspecific host switching) [6]. Feeding on different plants can be beneficial by improving nutrient balance or by reducing exposure to particular noxious compounds [1]. Highly polyphagous orthopterans are known to mix their diet to reach an optimal nutritional intake [7,8], and some generalist lepidopterans are thought to have a broad diet to improve their immune response [9,10]. Host switching can also be driven by necessity, for example if the original food source is depleted or represents a high risk of encounters with natural enemies [11]. Some insect herbivores have been shown to extend their normal dietary range during a population outbreak after depletion of their usual food sources [12]. Such circumstantial host switching may be detrimental to the fitness of the herbivore if the new host is a suboptimal species that would normally be avoided [13], but is obviously beneficial if the alternative is starvation and death. In the case of biological invasions of insect herbivores, dietary flexibility is often regarded as a trait facilitating both the establishment and spread of the exotic herbivore into new environments [14].

The viburnum leaf beetle, *Pyrrhalta viburni* (Paykull), is an invasive chrysomelid native to Eurasia and invasive in North America, where it was introduced in the 1920s in Canada, and is now considered a major landscape and forest pest in southern Canada and northeastern U.S. [15,16,17]. Both larvae and adults are dietary specialists restricted to plants in the genus *Viburnum* (Adoxacae, Dipsacales), a clade of small trees and shrubs of worldwide importance as ornamentals [18]. Egg is the overwintering stage; larvae emerge in the spring and go through three developmental instars before pupating in the soil. Adults emerge in early summer and typically remain in the field until August-September. In its introduced range, *P. viburni* is attacking populations of several native *Viburnum* spp. in natural habitats (e.g., forest understories, old-fields, and wetlands) and managed landscapes [19]. Susceptible shrubs attacked by larvae in the spring and adults in the summer often die after 2–4 years of repeated defoliations [17]. Within the *Viburnum* genus, several species are not optimal or unsuitable to the development of *P. viburni* larvae [20], and are avoided by adults for oviposition under natural conditions [21]. However, in mixed ornamental gardens containing both suitable and suboptimal host plants, *P. viburni* larvae have been observed, during population outbreaks, to resort to feeding on *Viburnum* hosts normally avoided after the complete defoliation of their favorite hosts (GAD personal observation [22]). Whether *P. viburni* larvae are able to complete their development after transfer on a suboptimal host is currently unknown. This information may help understanding the ecological context of *P. viburni* invasion in North America, and provide insight on cultural practices that could facilitate *P. viburni* management.

Here we investigated the effects of host switch for *P. viburni* larvae reared on an optimal host (*Viburnum dentatum* or *V. trilobum*, also known as *V. opulus* var. *americanum*) until the 3rd developmental instar and then transferred to a suboptimal host. We used three suboptimal host plants in this study: the Koreanspice viburnum *V. carlesii*, the Nannyberry viburnum *V. lentago*, and the Siebold viburnum *V. sieboldii*, and measured several insect fitness correlates (survivorship, pupal and adult weight) after transfer. In addition to this performance experiment, complementary feeding choice-tests were conducted with 3rd instar *P. viburni* larvae falling into two weight categories (<6 mg and >6 mg) and optimal and suboptimal host plants to answer the following questions: (1) do larvae exhibit feeding preferences between optimal and suboptimal hosts, and among suboptimal hosts? And (2) does the preference for certain host plants decrease as larvae get heavier and closer to pupation?

## 2. Experimental Section

### 2.1. Insect and Plant Material

Insects were maintained in vented plastic boxes (27.5 × 20 × 9.5 cm) in chambers held at 22 °C under a 16:8 (L:D) photoperiod. Larvae of *P. viburni* were obtained from *V. dentatum* twigs naturally infested with eggs of *P. viburni* collected in the field in the Ithaca, NY area the winter preceding the experiment. Egg-infested twigs were kept in a refrigerator until needed, and then transferred to an incubator maintained at 17 °C for three days, and finally to a chamber held at 22 °C until egg hatching. The foliage used for rearing the larvae until the 3rd developmental instar was kept in floral tubes (13 mm diameter × 10 cm).

Five species of *Viburnum* were used in this study, covering three categories of resistance to viburnum leaf beetle, as defined by Weston [23]: *Viburnum dentatum* and *V. trilobum* are highly susceptible (destroyed in the field after 2–3 years of infestation), *V. lentago* is moderately susceptible (shows variable degree of susceptibility but usually survives in the field), and *V. carlesii* and *V. sieboldii* are resistant (show very little or no damage in the field, survive infestations very well). *Viburnum dentatum*, *V. trilobum* and *V. lentago* are North American species, while *V. carlesii* and *V. sieboldii* are native to Asia. Previous research showed that *P. viburni* larvae develop optimally when reared on *V. dentatum* and *V. trilobum* [21], whereas *V. lentago*, *V. carlesii*, and *V. sieboldii* are suboptimal hosts for *P. viburni* when reared from egg hatch on these species [20,21]. All the plant material used for this study came from shrubs planted in the same field plot (Bluegrass Lane Turf Farm, Ithaca, NY, USA), and had been in the ground for at least 8 years. None of the plants used had a history of insecticidal treatment. Leaves were collected when needed by clipping branch terminals, and then held for no more than a day in a 22 °C (72 F) chamber until used for the bioassays.

### 2.2. Bioassays

We used Petri dishes (8.5 cm dia × 2.5 cm) with lids that had a portion of the plastic replaced with fine polyester netting (20 × 24 mesh/in) as bioassays arenas. A layer of 1 cm of moistened sand was put at the bottom of each Petri dish as a pupation substrate. The sand also helped the *Viburnum* leaves used during the experiment to remain fresh by providing moisture.

**Figure A1:**
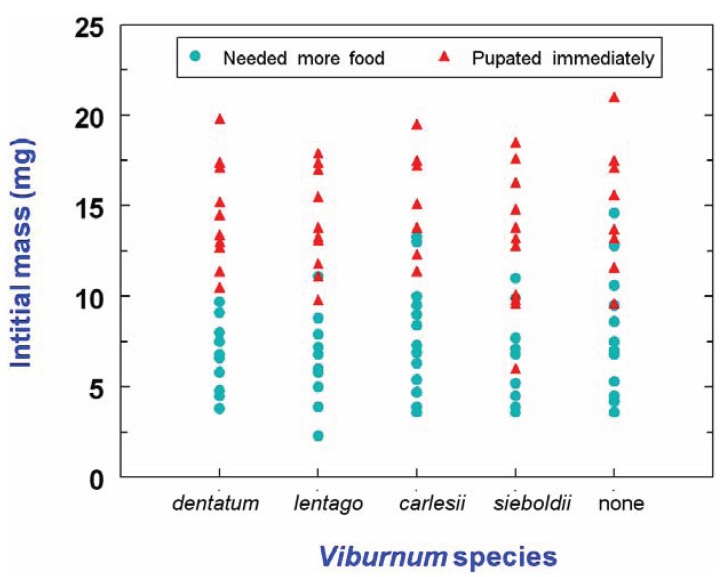
Distribution of the initial weight of individual *P. viburni* 3rd instar larvae used during the performance experiment. Color and shape of the dots indicate whether the larva pupated immediately by burrowing in the soil, or needed more food (*i.e.*, started to feed on the host presented or starved to death).

Larvae of *P. viburni* were reared in a single colony on fresh *V. dentatum* leaves collected in the field during the first two instars. Once they reached the 3rd instar (pre-pupal instar), 100 larvae were selected and weighed individually, then separated in 5 groups of 20. We ensured a similar weight distribution among treatments, from newly molted 3rd instar larvae to heavier larvae that had already fed and were closer to pupation (Figure A1). Each larva was then put into a bioassay arena dish with one of the diets tested: one leaf of *V. dentatum* (positive control), *V. lentago*, *V. carlesii*, or *V. sieboldii*, or with no food (negative control). Weight of larvae was recorded individually every day until death or pupation. Bioassay arenas were then monitored daily for adult emergence. Emerging adults were not given access to food and were weighed and sexed in the 12 h following emergence. Every larva was considered a single replicate, which provided a total of 20 replicates per treatment. 

In addition to larval, pupal, and adult weight, the amount of herbivory on the different diets was quantified by measuring the leaf area consumed by each larva at the end of the experiment. Leaf area consumed was quantified by comparing the leaf area remaining after feeding with the area of the same leaf as it had not been fed upon. Leaf areas were readily quantified using Scion Image, ver. 0.4 (Scion Corporation: Frederick, MD, USA) after leaf scans were converted to black and white bitmap images using PhotoShop ver. 5.0 (Adobe Systems, Inc.: San Jose, CA, USA). Because scans of the leaves were not made before the experiment, the area of the intact leaf had to be estimated by manually filling in the leaf area consumed using a drawing tool in Scion Image. Consumption was then estimated by subtracting the leaf area after feeding from the area of the reconstructed (virtual) intact leaf. Areas in Scion image were then converted from pixels to cm^2^ by multiplying by the appropriate conversion factor obtained from a scan of a leaf disk of known size (1 cm diameter).

The efficiency of conversion of ingested food (ECI) was also calculated for each larva at the end of the experiment by dividing the total weight gained (*i.e.*, sum of the daily weight gains until pupation) by the total weight of food consumed. Weight of the food consumed was quantified by converting the leaf area consumed into weight using the appropriate conversion factors. Conversion factors were determined separately for each *Viburnum* species by measuring the total leaf area of five leaves of known weight.

To complement the insect performance experiment, feeding choice-tests were conducted with 3rd instar *P. viburni* larvae. We divided larvae that had been reared on an optimal host in two categories depending on their weight: larvae with a weight less than 6 mg and larvae with a weight over 6 mg. Larvae under 6 mg correspond to newly-molted larvae, while larvae over 6 mg are likely to have already fed during the 3rd instar, and to be thus closer to pupation. Choice-tests were conducted in vented rectangular plastic boxes (27.5 × 20 × 9.5 cm) by giving the choice to 20 larvae between the optimal host and a suboptimal host, or between two suboptimal hosts. One shoot of each of the two test plant species was placed on each side of the box, and larvae were deposited in the center of the box (plant shoots were placed in floral tubes to retain moisture). The number of larvae present on each plant was recorded 24 h after the start of the experiment. We made the assumption that larvae would keep wandering in the test arena until they find a host plant they accept and start feeding on, and that a 24 h period was long enough for the larvae to encounter both host plants present in the arena (unless they accepted the first host plant they encountered). Larvae that failed to make a choice (*i.e.*, were not present on a plant after 24 h) were counted and discarded from the analysis of the results. Three replicates of the choice-test were conducted for each pair of host species tested and each larval weight category. The suboptimal hosts that were used for the choice-test experiment were the same as for the performance experiment: *V. lentago*, *V. carlesii*, and *V. sieboldii*. No 3rd instar *P. viburni* larvae reared on *V. dentatum* were available at the time of the experiment: the optimal host used for this experiment was the North American *V. trilobum.* Previous studies showed that *P. viburni* performs equally well on *V. dentatum* and *V. trilobum* [21,24]. Larvae used for the choice-tests had been reared from egg hatch to the 3rd instar on *V. trilobum*.

### 2.3. Statistical Analysis

The effects of insect diet on larval and pupal weight, daily weight gained, total herbivory, and ECI data were analyzed separately using analysis of variance (ANOVA). Daily weight gain values were averaged for each larva in each treatment to have only one data point per larva in the model. Means were compared with the LSD all-pairwise comparison procedure (Statistix ver. 8.0, Analytical Software: Tallahassee, FL, USA). Daily weight gained data were cubic-root transformed to meet the assumptions of the ANOVA model. The effects of sex and larval diet on adult weight were analyzed using the analysis of variance (ANOVA) model (Statistix ver. 8.0, Analytical Software: Tallahassee, FL, USA).

To analyze the results of the choice-tests, an analysis was performed to determine whether larvae became less choosy as they got closer to pupation. For this analysis, a “choosiness” dependent variable was calculated as the difference between larvae that chose species A and the larvae that chose species B after 24 h for each choice-test. Species A was defined as the species where the majority of the larvae were found, and species B was the species where the minority of larvae were found. Thus, the variable choosiness was a positive value, and the value for choosiness increased as larvae made more marked choices. We also defined the variable “test type” as a categorical variable with two levels, depending on which hosts were included in the test: optimal *vs*. suboptimal, and suboptimal *vs*. suboptimal. An ANOVA was performed testing the effects of test type, larval weight, and the interactions between both terms on choosiness.

## 3. Results and Discussion

### 3.1. Direct Pupation and Acceptance of the Diet

Among the 100 larvae monitored individually until death or pupation on the different treatments tested, 46 pupated less than 24 h after the beginning of the experiment (Figure A1). These larvae did not feed on the leaf material and directly burrowed into the sand to pupate. The average weight of these larvae was 14.2 ± 1.6 (mean ± SE) mg, and the average initial weight of larvae that pupated directly was not significantly different among diets (one-way ANOVA, F_4,41_ = 0.7, *p* = 0.6). Larvae that pupated immediately were excluded from further data analysis. Among the remaining larvae (54 individuals), 95.2% started to feed on the leaf material. The rest of the larvae, including those that were given no food (negative control), starved and quickly died.

### 3.2. Effect of Diet on Survivorship, Weight Gained, ECI, and Pupal and Adult Weight

**Figure 1:**
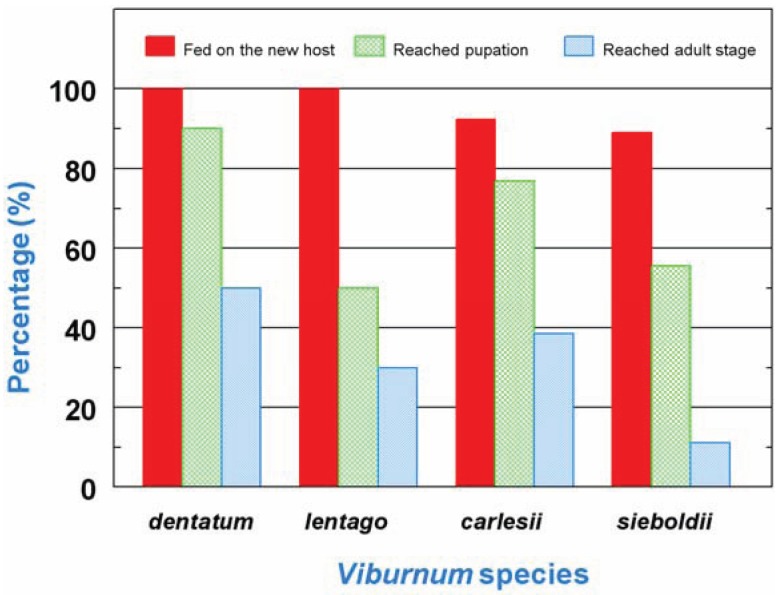
Development of *P. viburni* 3rd instar larvae after transfer from an optimal host species (*V. dentatum*, n = 10) to the same host or to a suboptimal host (*V. lentago*, n = 10; *V. carlesii*, n = 13; *V. sieboldii*, n = 9). The mean duration of development until adult emergence was 30.0 ± 2.4 days.

All of the larvae that were given *V. dentatum* leaves as food (= positive control) fed on the new plant material and 90% reached the pupal stage. The majority of the larvae that were given a suboptimal species fed on the new diet (from 88.9% on *V. sieboldii* to 100% on *V. lentago*) and showed varying levels of success at reaching the pupal stage (from 50% on *V .lentago* to 76.9% on *V. carlesii*). Larvae in all the treatments experienced some degree of pupal mortality, from 20% on *V. lentago* to 44.5% on *V. sieboldii*, but at least a small percentage of the starting larvae reached the adult stage, from 11.1% on *V. sieboldii* to 50% on *V. dentatum* (Figure 1). Out of the 54 larvae that fed on the new diet, 18 reached the adult stage: 8 on *V. dentatum*, and 10 total on the suboptimal hosts. The sex ratio male:female of adults originating from the *V. dentatum* diet and from the suboptimal diets were comparable (5:3 and 6:4, respectively).

**Figure 2:**
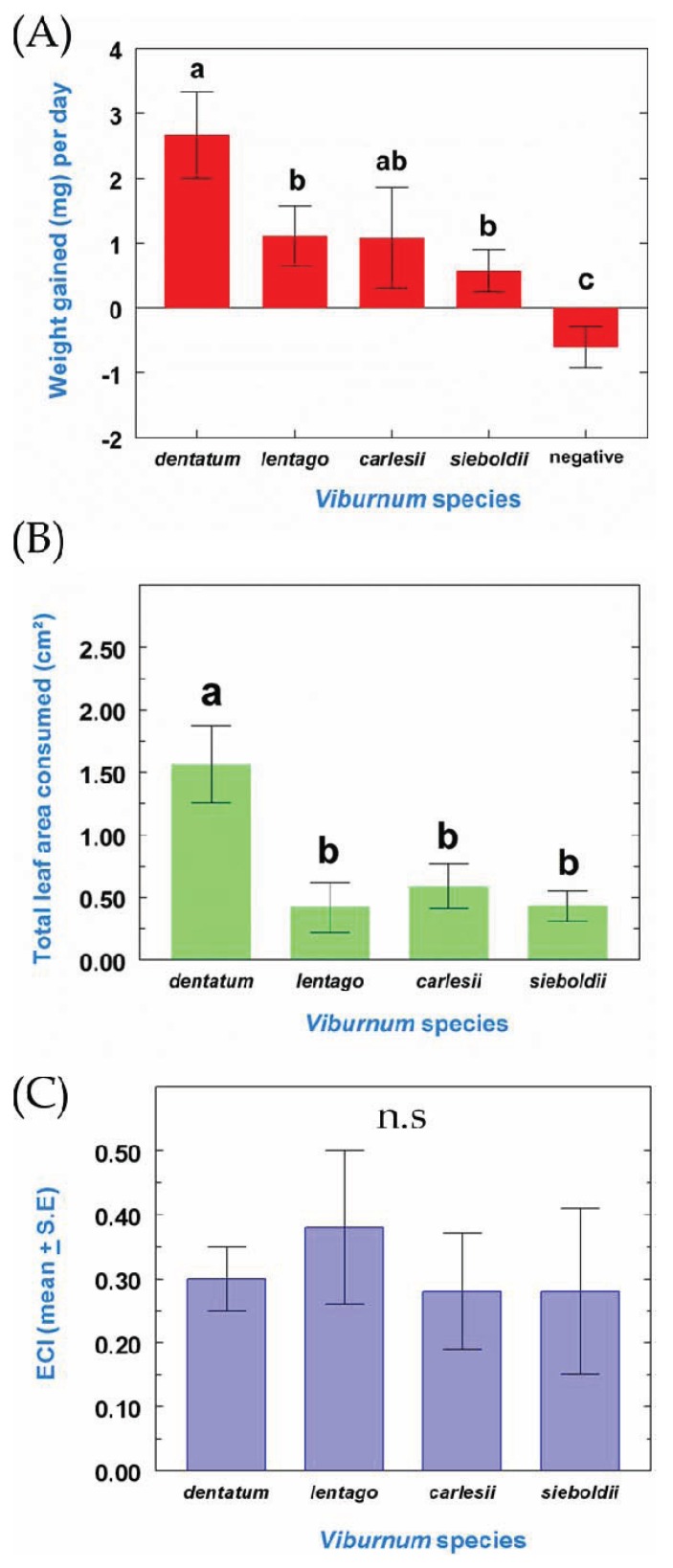
(**A**) Daily weight gained (mg), (**B**) total leaf area consumed, and (**C**) efficiency of conversion of ingested food (ECI) (*i.e.*, total weight gained/total weight ingested) by *P. viburni* 3rd instar larvae after transfer from an optimal host species (*V. dentatum*) to the same host or to a suboptimal host (*V. dentatum*, n = 10; *V. lentago*, n = 10; *V. carlesii*, n = 13; *V. sieboldii*, n = 9) (mean ± SE). In the “negative” treatment (**A**), *P. viburni* larvae were not provided any food. Means followed by a different letter are statistically different (One-way ANOVA, LSD all-pairwise comparisons, *p* < 0.05). n.s. = non-significant.

There was a significant difference in daily weight gained per larva among treatments (F_4, 46_ = 16.7, *p <* 0.0001). Daily weight gained was highest on *V. dentatum,* intermediate on *V. carlesii*, lower on *V. lentago* and *V. sieboldii*, and lowest for the negative control treatment. When given no food, larvae did not gain any weight, and lost an average of 0.6 ± 0.3 (mean ± SE) mg per day (Figure 2A). There was a significant effect of diet on herbivory (F_3,34_ = 14.5, *p* < 0.0001), with larvae consuming more *V. dentatum* leaves than the other host species (Figure 2B). However there was no effect of the diet on the ECI ratio (F_3,34_ = 0.5, *p* = 0.7); ECI values ranged from 0.28 for *V. carlesii* to 0.38 for *V. lentago* (Figure 2C).

**Figure 3:**
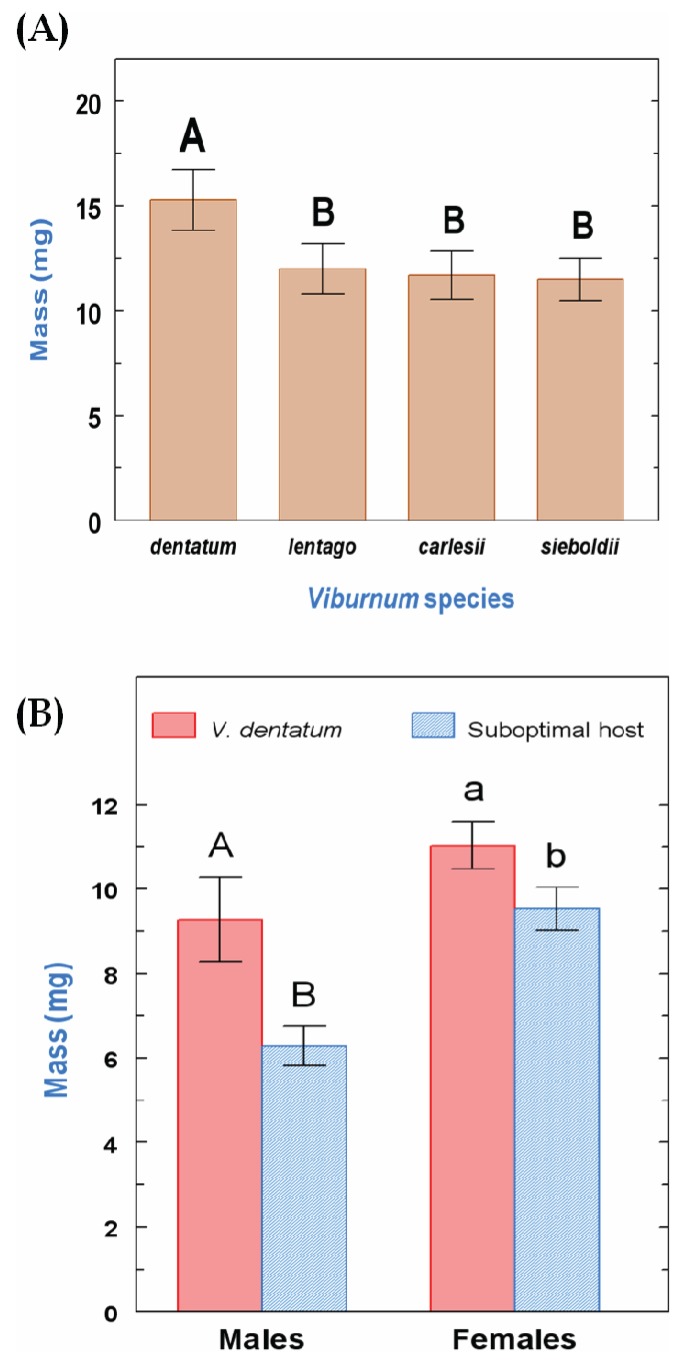
(**A**) Pupal weight and (**B**) adult weight reached by *P. viburni* 3rd instar larvae after transfer from an optimal host species (*V. dentatum*) to the same host or to a suboptimal host (*V. dentatum*, n = 10; *V. lentago*, n = 10; *V. carlesii*, n = 13; *V. sieboldii*, n = 9) (mean ± SE). Means followed by a different letter are statistically different (one-way ANOVA, LSD all-pairwise comparison, *p* < 0.05).

Weight reached at pupation was significantly different among treatments (F_3,27_ = 4.9, *p* < 0.01), and was higher when larvae were fed *V. dentatum* leaves than one of the suboptimal diets (Figure 3A). There was an effect of both larval diet and sex on adult weight (F_1,14_ = 13.5, *p* < 0.01 and F_1,14_ = 16.5, *p* < 0.01, respectively), but no effect of the interaction between both factors: adult weight was higher for females than for males, and when larvae were fed *V. dentatum* leaves than leaves of a suboptimal host (Figure 3B). Due to the low number of larvae that reached the adult stage, analysis of differences in adult weight among suboptimal host species was not possible.

### 3.3. Feeding Choice-Tests

**Figure 4:**
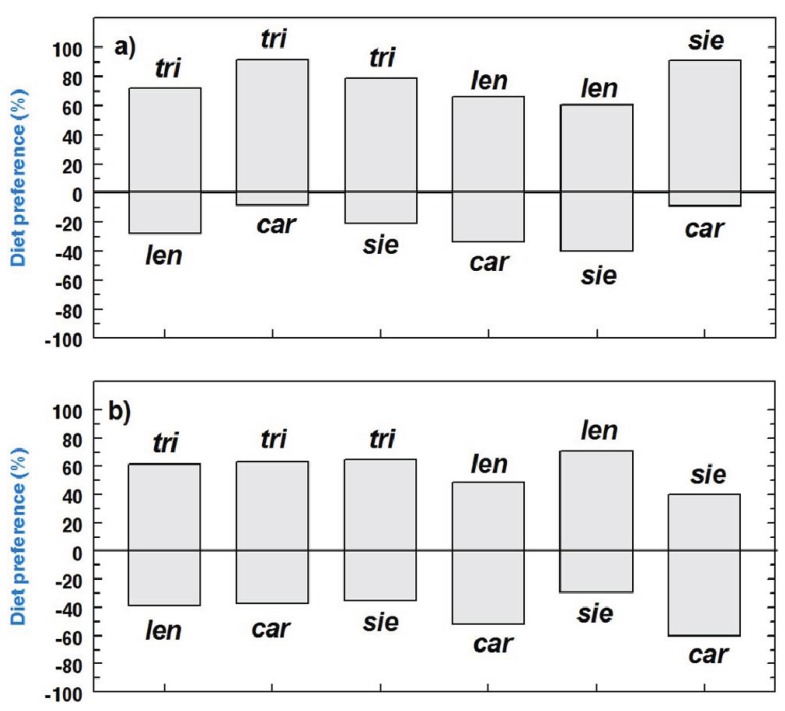
Feeding preferences of *P. viburni* 3rd instar larvae among the four following *Viburnum* species: *V. trilobum* (*tri*), *V. lentago* (*len*), *V. carlesii* (*car*), and *V. sieboldii* (*sie*) for larvae weighing (**a**) less than 6 mg, and (**b**) more than 6 mg. Y-axis indicates the percentage of larvae that chose each host for each pair of species tested (n = 3 for each host pair).

Out of 20 larvae placed in the arena for each choice-test, 1.4 ± 0.3 did not make a choice after 24 h, and 0.8 ± 0.2 died (grand mean for all species ± SE). The rest of the larvae were found on one host or the other. Overall, when given the choice between the optimal host *V. trilobum* and a suboptimal host, 72.6% of the larvae chose the optimal host (262 out of 361 larvae). When given the choice between suboptimal hosts, 58.8% of the larvae preferred *V. lentago* over *V. carlesii* (59 out of 101 larvae), 69.8% preferred *V. sieboldii* over *V. lentago* (60 out of 86 larvae), and 66.1% preferred *V. sieboldii* over *V. carlesii* (74 out of 112 larvae) (Figure 4). The choosiness of *P. viburni* larvae, as defined as the difference between the most preferred host and the least preferred host for each choice-test, was dependent on larval weight (F_1,33_ = 8.9, *p* < 0.01), but not on the test type (optimal *vs*. suboptimal or suboptimal *vs*. suboptimal) (F_1,33_ = 2.6, *p* = 0.1) nor the interaction between both terms (F_1,33_ < 0.1, *p* = 0.8). Larvae were more choosy and had more marked preferences when they were lighter (<6 mg) than when they were heavier (>6 mg) (Figure 4).

### 3.4. Discussion

Switching from a host plant species to another one during immature development can have significant costs to the fitness of phytophagous insects, especially if the insect has developed an acquired preference for its initial diet [25,26]. However, dietary flexibility can also be a life-saving strategy if the preferred food sources of the insect get depleted, which can occur for instance during population outbreaks.

Our study shows that *P. viburni* 3rd instar larvae are negatively affected by the transfer from an optimal host (*V. dentatum*) to a suboptimal host; although at least a small percentage of larvae managed to reach the adult stage after transfer on each of the suboptimal hosts tested (Figure 1), pupal and adult weight were consistently lower after transfer compared to larvae that were kept on *V. dentatum* during their entire development (Figure 3). This weight decrease appears as a direct result of reduced feeding rates on the suboptimal hosts, whereas the efficiency of conversion of ingested food remained consistent across all the diets tested (Figure 2). These results mean that the suboptimal hosts tested are not poorer nutritionally than *V. dentatum*, but the larvae were limited by the quantity of food they were able to ingest on a daily basis on the suboptimal hosts. Reduced feeding rates may originate from the presence of secondary metabolites in the suboptimal hosts that function as feeding inhibitors or from reduced feeding stimulation on these diets [3,27]. Interestingly, *V. carlesii*, despite being considered resistant to *P. viburni*, seemed to be more suitable for host switching than the moderately susceptible *V. lentago* (Figure 1). It is possible that *V. carlesii* is more resistant to young *P. viburni* larvae feeding from egg hatch than to 3rd instar larvae [20].

One complicating factor that our study does not address is the recently reported observation that *P. viburni* benefits from larval group feeding [28]. Although *P. viburni* larvae do not feed gregariously in the strict sense [29], they do tend to form loose groups feeding on the undersides of young Viburnum leaves, a behavior that is facilitated by the fact that egg masses from adult *P. viburni* are often laid in clusters along infested twigs [30]. Larval density was shown to be positively correlated with pupation success and, under certain conditions, adult weight on a range of Viburnum hosts [28]. Thus, larval density may play a role in the success of host-switching in *P. viburni* and, conversely, results of the choice-tests could have been different if larvae had been tested individually. This factor could be particularly relevant in the case of population outbreaks, with larvae emigrating en masse to less preferred host plants after depletion of their favored food sources. Benefits of larval group feeding to overcome plant defenses have been documented in other systems, including lepidopteran caterpillars [31] and aphids [32].

Not too surprisingly, *P. viburni* larvae showed consistent preferences for the optimal host in feeding choice-tests (Figure 4), indicating a strong preference-performance relationship that had already been evidenced in oviposition preferences of *P. viburni* females [21]. Feeding preferences among suboptimal hosts were less clear and consistent, although *V. sieboldii* seemed to be the most preferred suboptimal host: 67.7% of the larvae (134 out of 198) chose *V. sieboldii* when in a choice-test with another suboptimal host (Figure 4). Overall, feeding preferences were stronger for young 3rd instar larvae (<6 mg) than for older larvae (>6 mg), indicating that larvae become less choosy as they approach the end of their development and thus become more likely to accept a suboptimal host (although becoming less choosy does not necessarily mean they will more likely to successfully develop on a suboptimal host). The fluctuations of insect dietary flexibility throughout immature development are a theme that has been barely explored in plant-insect interactions [3] and nutritional ecology [33], but could have some important ecological implications. Testing the effects of host switch and preferences of *P. viburni* larvae with optimal hosts only would be an interesting development of this study.

## 4. Conclusions

In summary, *P. viburni* larvae are able to complete their development after being transferred to a less preferred, suboptimal host, but this host switch has a general negative effect on fitness correlates (pupal and adult weight). Under natural conditions, transfer to suboptimal hosts may be facilitated by the fact that larvae become less choosy toward the end of their development. In its introduced range, *P. viburni* is an outbreaking herbivore that commonly completely defoliates its preferred host plants in managed landscapes and under natural conditions. Mixed Viburnum gardens containing both susceptible (preferred) and resistant (suboptimal) Viburnum species growing in close proximity provide opportunities for *P. viburni* to survive larval outbreaks by giving them secondary hosts to transfer to once their favored food sources have been depleted. Thus, we recommend Viburnum growers and landscape managers avoid planting susceptible and resistant Viburnum species in close proximity to limit the risks of persistence of *P. viburni* populations following a larval outbreak.
